# COMMON NONCARDIOVASCULAR MULTIMORBIDITY PATTERNS AND OUTCOMES IN OLDER ADULTS WITH MAJOR CARDIOVASCULAR DISEASE

**DOI:** 10.1093/geroni/igac059.2450

**Published:** 2022-12-20

**Authors:** Stephanie Denise Sison, Joshua K Lin, Mehdi Najafzadeh, Elisabetta Patorno, Lily G Bessette, Heidi Zakoul, Dae Kim

**Affiliations:** Beth Israel Deaconess Medical Center, Brookline, Massachusetts, United States; Brigham and Women’s Hospital, Boston, Massachusetts, United States; Brigham and Women’s Hospital, Boston, Massachusetts, United States; Brigham and Women's Hospital, Harvard Medical School, Boston, Massachusetts, United States; Department of Medicine, Boston, Massachusetts, United States; Brigham and Women’s Hospital, Boston, Massachusetts, United States; Hebrew SeniorLife, Boston, Massachusetts, United States

## Abstract

Non-cardiovascular multimorbidity often coexists in older adults with cardiovascular disease (CVD), but their clinical significance is uncertain. Using Medicare data, we examined common non-cardiovascular comorbidity patterns and their association with clinical outcomes in beneficiaries with congestive heart failure (CHF), acute myocardial infarction (AMI), or atrial fibrillation (AF). We created 3 CVD cohorts of beneficiaries diagnosed with CHF (n=57,285), AMI (n=24,808), and AF (n=36,277) before January 1, 2016. Within each cohort, we applied latent class analysis to classify beneficiaries based on 7 non-cardiovascular comorbidities, including dementia, cancer, chronic kidney disease (CKD), chronic lung disease, depression, diabetes, and musculoskeletal disease. Mortality, cardiovascular and non-cardiovascular hospitalization, and home time lost until December 31, 2016 were compared across non-cardiovascular multimorbidity classes. Similar non-cardiovascular multimorbidity classes emerged from 3 CVD cohorts: 1) minimal, 2) depression (or lung-depression in AMI cohort), 3) diabetes-CKD, and 4) multi-system class. Compared to minimal class, multi-system class had the highest risk of mortality (hazard ratio [HR], 3.0 to 3.7), cardiovascular hospitalization (HR, 1.6 to 3.4) and non-cardiovascular hospitalization (HR, 2.8 to 6.0), and home time lost (rate ratio, 2.8 to 4.8), followed by lung-depression/depression or diabetes-CKD classes. In CHF and AF cohorts, multimorbidity classes were associated with greater increase in non-cardiovascular hospitalizations than cardiovascular hospitalizations. Our findings emphasize that improving health of older adults with CVD requires attention to non-cardiovascular multimorbidity.

